# Thermal Time Constant CNN-Based Spectrometry for Biomedical Applications

**DOI:** 10.3390/s23156658

**Published:** 2023-07-25

**Authors:** Maria Strąkowska, Michał Strzelecki

**Affiliations:** Institute of Electronics, Lodz University of Technology, 93-590 Lodz, Poland; michal.strzelecki@p.lodz.pl

**Keywords:** CNN, active thermography, biomedical application, thermal time constants, deep learning, noisy signals

## Abstract

This paper presents a novel method based on a convolutional neural network to recover thermal time constants from a temperature–time curve after thermal excitation. The thermal time constants are then used to detect the pathological states of the skin. The thermal system is modeled as a Foster Network consisting of R-C thermal elements. Each component is represented by a time constant and an amplitude that can be retrieved using the deep learning system. The presented method was verified on artificially generated training data and then tested on real, measured thermographic signals from a patient suffering from psoriasis. The results show proper estimation both in time constants and in temperature evaluation over time. The error of the recovered time constants is below 1% for noiseless input data, and it does not exceed 5% for noisy signals.

## 1. Introduction

Time-constant spectrometry is a method of reducing the dimensionality of data and signals describing time-varying dynamic processes. This technique can be used for various engineering and medical applications. Initially, this method was successfully applied for dynamic object identification, mainly in the fields of automation and control [1,2,3,4,5,6,7,8] and electrical and electronic engineering [9,10,11,12]. Thermal time-constant spectrometry is a method that effectively reduces the dimensions of the mathematical representation of a problem. It approximates a large dataset in the time and frequency domains by using a series with only a few components. This technique has already been employed in the modeling of long energetic lines to predict their performance [12]. The distribution of time constants finds application in communication system modeling, utilizing the immittance concept [4]. Additionally, time-constant spectrometry can simplify the modeling of a complex electrical network in dynamic states, aiding in the analysis and prediction of its behavior [2]. Recently, time-constant spectrometry has also been used for biomedical applications where the skin temperature is monitored by infrared (IR) thermography [13,14]. In the heat transfer domain, thermal systems can be modeled as the R-C Foster Network that are directly corresponding to the time-constant distribution [9,10,13,14,15,16,17]. R-C networks are used for thermal object characterization. Such a network is a chain of thermal resistance and capacitance connections. Each element of the ladder is the parallel connection of thermal resistances and thermal capacitances, while all cells are connected in series. Such modeling is the result of a multilayer structure where each of the branches corresponds to each of the layers. 

It has to be underlined that the process of time-constant identification of a dynamic system is a kind of inverse problem that is, in general, ill-conditioned. In particular, it is a very severe problem in the heat transfer domain because the non-orthogonal set of functions approximates the temperature changes in the dynamic processes. 

There are a few existing methods being used for dynamic system identification based on data processing in either the time or frequency domain. In electronics for thermal problems, network identification by deconvolution (NID) is widely used [9,10,11]. In electrical engineering, continuous-time system identification (CONTSID) [2,3] or computer-aided program for time-series analysis and identification of noisy systems (CAPTAIN) [17,18] are known. Typically, these methods are based on the rational presentation of the transfer function in frequency domain or a polynomial approximation of the impulse response of a system in time domain. A very practical and widely used software tool for dynamic system identification is offered in the Matlab environment. It uses built-in transfer function estimation (TFEST) [13,14]. Recently, a new approach has been implemented using the Vector Fitting algorithm for inverse heat transfer problem solution [12,13,14,15,16,17,18,19]. It is important to mention that in the biomedical sciences, screening and diagnostic procedures must be non-invasive and, in many cases, contactless. That is the reason that IR thermography is now more and more useful [13,15]. 

Despite the problem of ill-conditioning, the signals measured by IR techniques have low amplitude, are noisy, and are disturbed by various unstable environmental effects.

Therefore, the learning system is proposed to solve the inverse problem [20]. In fact, this system simply realizes non-linear regression to approximate a set of multidimensional functions [21,22]. For such a problem, artificial intelligence methods are suitable, including deep learning approaches. In this research, the convolutional neural network (CNN) is proposed. To our knowledge, this is a novel approach for approximating dynamic temperature curves. It is known that in order to apply CNNs successfully, the training data must be large and reliable. In general, it is rather difficult to provide such training knowledge. Therefore, transfer learning is often included. It uses both data from different modalities and data artificially generated using appropriate modeling [20]. There are different CNN models that exist that are used both for classification and regression purposes [20,21]. Almost all of them implement the concept of residual networks with long or short skip connections, known as the ResNet architecture [20,21]. The approach is useful when the training and validation data do not differ much. Otherwise, it can lead to gradient vanishing during the learning optimization process [23,24,25]. This may regard heat transfer inverse problems as very ill-conditioned. In practice, it means that a large variation in the input will produce a small change in the output, and vice versa. 

There are numerous studies exploring the application of AI in the field of thermovision for biomedical purposes. Most of these studies use CNN networks for segmentation and classification tasks. In reference [26], the authors present software capable of classifying thermal images of neonates as healthy or unhealthy and visualizing the skin regions that contribute to this classification. CNN is used to classify non-alcoholic fatty liver disease by extracting texture features from thermal images [27]. CNN networks can also be used to segment patients’ breasts in thermal images and classify pathologies [28]. Reference [29] demonstrates the results of using CNN networks to classify thermograms of healthy and arthritis-affected knees. All these applications demonstrate the potential of deep learning algorithms in biomedical applications based on thermal imaging. CNN can also be used for regression tasks, e.g., in dynamic thermovision applications. 

The aim of this work is to verify whether it is possible to apply the CNNs to thermal time-constant spectrometry. The proposed approach has been verified both on artificial data and on measurements performed on patients with psoriasis. The distribution of time constants, which illustrates the temporal response of the tissue to thermal stimulation, is used to distinguish between its pathological and physiological states.

The rest of this document is organized as follows:

– Section 2: Materials and Methods—presents the proposed approach for the time-constant spectrometry method based on CNN. It includes a description of the proposed structure of the CNN network, the dataset, and the training and validation processes.
– Section 3: Results—is divided into subsections:
  ○ Section 3.1—presents the results of network validation for artificially generated data.
  ○ Section 3.2—presents the results for artificially generated data with different levels of noise added.
  ○ Section 3.3—presents the network verification for real measurements of the patient suffering from psoriasis—for healthy and unhealthy parts of the skin.
– Section 4—discusses the results, concludes the paper, and lists some possible future work.

## 2. Materials and Methods

Time-constant spectrometry requires solving the ill-conditioned inverse problem. In this research, a regressive convolutional neural network is proposed for this purpose. The problem is to reconstruct the thermal time-constant distribution from the temperature changes over time, measured using a high-speed thermal imaging camera. Temperature curves can be approximated by the sum of a few exponential components, each representing a thermal time constant [30]. Tests show that a four-time-constant approximation gives satisfactory results with a low value of error between the original and predicted data. This number of time constants was chosen for the training procedure.

**Figure 1:**
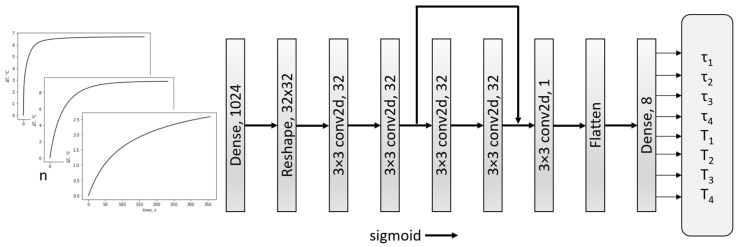
CNN network structure.

Several convolutional neural networks with and without residual layers were tested. In addition, different numbers of feature layers, filters used, and kernel sizes were implemented, and their effectiveness was verified. The software was developed in the Python environment using the TensorFlow and Keras libraries [31]. After several attempts, the network shown in Figure 1 was selected.

The network consists of nine layers. The first and last layers are dense. They transform input and output into lower- and higher-dimensional representations, respectively. The convolutional layers in between use 32 filters with a kernel size of 3 × 3 and apply the sigmoid activation function.

The convolutional layers perform the 2D convolution operations on the input data, extracting features using the 32 filters. The ‘padding’ parameter is set to ‘same’ to preserve the spatial dimensions of the output feature maps. The last convolutional layer has a single filter and produces a single-channel output.

This output is then connected to a Flatten layer, which reshapes it into a 1D vector with 1024 elements. The flattened data are passed through a dense layer with 8 neurons, applying a linear transformation followed by the sigmoid activation function. This layer is responsible for generating the final output of the network, consisting of 4 thermal time constants and their amplitudes. 

Transfer learning was applied in this research. This means that the input data used for training the CNN network consists of curves that are generated artificially as the sum of four exponential components with known values of the amplitudes of the thermal time constants (Equation (1)). The thermal time constants and their amplitudes are randomly generated within the [0, 1] range, as shown in Table 1. In the presented research, each curve is composed of four exponential components.

(1) $$T\left(t\right)=\sum_{i=1}^{4}T_{i}(1-e^{-\frac{t}{\tau_{i}}})$$

The CNN model is designed to fit temperature rise measurements over time that are recorded by the IR camera at a frequency of 50 Hz for approximately 6 min. Each measurement consists of approximately 18,000 samples. Training the CNN network with thousands of such large data vectors can be time-consuming and requires significant memory and computing power. Furthermore, the crucial part of the temperature rise curve with the most important information about the time constants is at the beginning. To recover the dynamic behavior of the thermal system, the temperature curves over time are non-uniformly re-sampled, with denser sampling at the beginning and sparser sampling in the quasi-steady state region. To speed up the learning process, each temperature curve is represented by 1024 non-uniformly distributed samples only.

For training and validation, the ranges of the parameter values *τ_i_*, *T_i_* are normalized, i.e., *τ_i_* ∈ [0, 1] s, *T_i_* ∈ [0, 1] °C. To ensure data consistency during the test, the temperature evolution computationally generated in any time interval is scaled to [0, 1] s. A similar operation is performed for the temperature curves of the measured signals. Finally, both the thermal time constants and their amplitudes were scaled up using the same scaling coefficients. All these normalization operations essentially help in the CNN learning process. 

The main parameters of the proposed network are listed in Table 1.

The learning process uses a set of 10,000 training and 1000 validation data points, respectively. The input data consist of temperature curves over time calculated using the randomly generated time constants and their amplitudes. The entire training procedure takes 5000 epochs.

**Figure 2:**
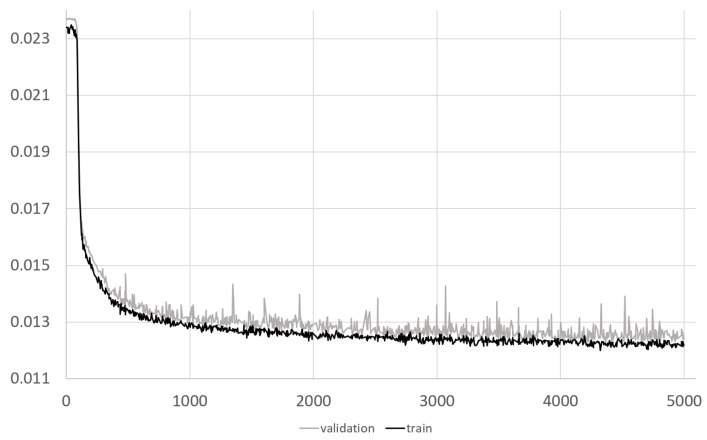
Loss function for train and validation data.

An example of the decay of the loss function values during training and validation is presented in Figure 2.

The trained network was tested on 1000 smooth curves, 1000 noisy curves, and 2 temperature measurements over time. The proposed method was also verified on a psoriasis patient, where both healthy and diseased parts of his skin were analyzed.

The loss function defined in the developed CNN network uses a weighted sum of the mean squared error of *τ_i_* and *T_i_*. The research results show that due to the greater impact of time constants compared to thermal amplitudes on the evolution of temperature in time, the network approximates *τ_i_* better than *T_i_*. Therefore, the effect of time constants on the loss function is arbitrarily reduced by defining the weighted loss function, as shown in Table 1.

The proposed CNN architecture is a compromise between its computational complexity and its performance. The loss function in Figure 2 confirms that the network still has the potential to learn, and we do not observe any signs of overlearning. It appears that there is room for larger datasets to further reduce the overall error of the network. The concept of the ResNet network with long connections accelerates learning and protects against gradient vanishing. Additionally, more advanced augmentation techniques can be applied.

## 3. Results

### 3.1. Network Validation for Artificially Generated Data

The training and validation data consist of the sum of exponential functions with four components. Time constants and amplitudes are randomly generated to produce these curves. The mean squared errors for 1000 samples show the deviation of the original and predicted parameters (*τ_i_* and *T_i_*). Additionally, the temperature curves over time are calculated for scaled data ranging from 0 to 1 to verify the effectiveness of the data processing. The results are presented in Table 2.

**Figure 3:**
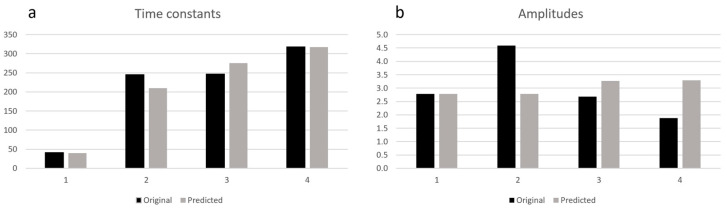
Exemplary original and predicted distribution of time constants (**a**) and amplitudes (**b**).

The mean value of MSE for the time constants does not exceed 1% (Table 2). Figure 3 presents the example result of the original and predicted unscaled values of *τ_i_* and *T_i_*. 

**Figure 4:**
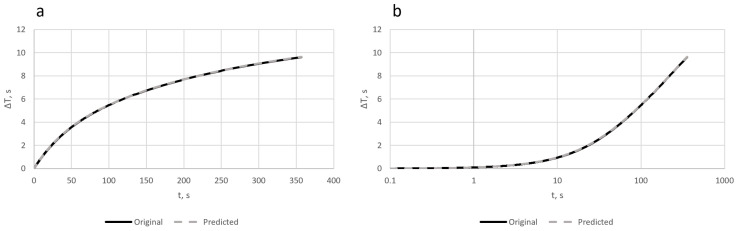
Original and predicted curves of temperature in time in linear (**a**) and logarithmic scales (**b**).

The temperature changes in time after upscaling to a 6 min of acquisition interval are presented in Figure 4.

### 3.2. Network Validation for Noisy Data

When using active thermography in medical applications, one must determine the temperature rise over time in a specific region of interest, typically a small spot. This task is challenging due to patient motion caused not only by controlled movements but also by physiological activities such as breathing and heartbeat. Therefore, motion correction techniques need to be applied [13,14]. This is a demanding task due to the low thermal contrast of IR images and the small size of the region of interest that needs to be recovered in each frame. Additionally, if the region of interest is located on the leg or arm, the rotation of these body parts can deform the spot and introduce errors in the temperature readings. Consequently, this can result in noise in the temperature curve over time.

The proposed method for extracting thermal time constants and their amplitudes was also tested on noisy data. A Gaussian noise with a zero mean and various variance values was added to the generated data. Mean squared errors were estimated for each test, which was performed on 1000 samples. The results are presented in Table 3. 

Obviously, the errors increase with higher values of the noise variance, but they do not exceed the levels of 5% and 9% even for the highest tested noise, both for time constants and their amplitudes, respectively (Table 3). This confirms that the proposed method is effective and can be used for real measurements that are often disturbed by noise.

**Figure 5:**
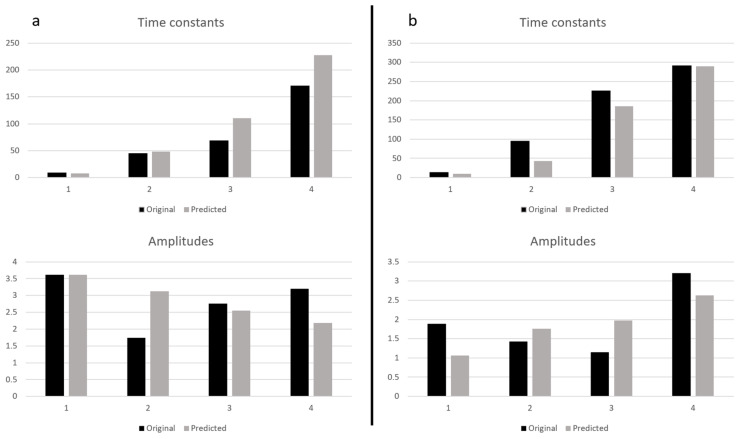
Exemplary original and predicted distributions of time constants and amplitudes for noisy data: noise variance 0.0001 (**a**) and 0.005 (**b**).

**Figure 6:**
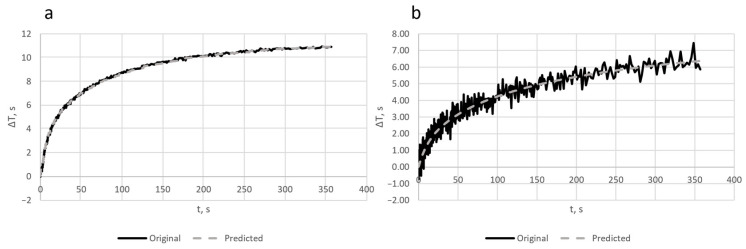
Original noisy signal and predicted curve of temperature in time for the noise with the variances of 0.0001 (**a**) and 0.005 (**b**).

The exemplary results of time constants and the amplitude distributions recovered for the noisy signals with the highest and lowest variance are presented in Figure 5, while the function of temperature in time is shown in Figure 6. The dense sampling at the beginning results in more frequent noise in this part of the curve. This simulates a more challenging problem to solve, leading to higher errors in such cases.

The visualization of the results for the lowest (a) and highest (b) values of the noise variances is presented in Figure 5.

The level of noise added to input data for the variances 0.0001 and 0.005 is clearly visible in Figure 6. 

### 3.3. Results for a Real Data

**Figure 7:**
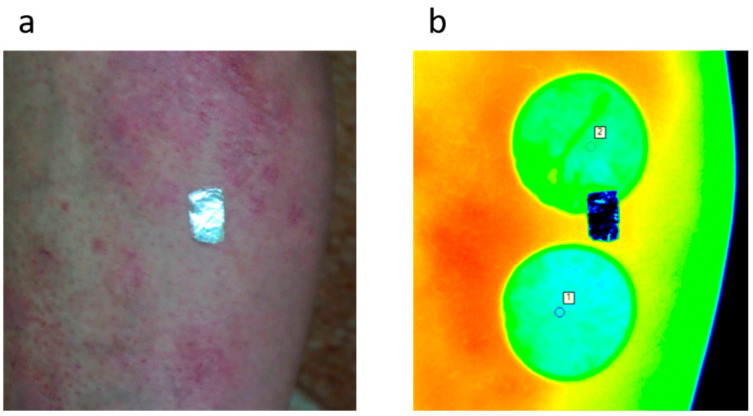
Photo of the patient’s leg with psoriasis (**a**) and its thermal image after thermal excitation with marked ROIs for the measurements (**b**). Warmer colors mean higher temperature.

The presented method could also be used for real measurement data, especially to determine the thermal time constants for living tissues. This would help to distinguish differences in skin reactions between healthy and unhealthy skin. Exemplary tests were made for the measurements carried out on a patient with psoriasis (see Figure 7). 

The thermographic examination was performed dynamically using so-called thermal stress. The skin on the patient’s leg was cooled down by metal blocks with a high thermal capacity by applying them to the patient’s skin for 5 s. Then the temperature rise over time was recorded for two regions of interest: one corresponding to healthy skin and the other to the psoriasis area. The size of the marked ROIs is 5 × 5 pixels. The temperature drop after cooling was approximately 5–6 °C. The measurement was performed for about 6 min to reach a quasi-steady state. 

A CEDIP Titanium camera with a cooled detector of 640 × 512 pixels was used to carry out the research. The use of this type of camera was necessary due to the continuity of the signal recording, unlike cheaper and smaller microbolometer cameras. The sequence of thermograms was recorded at a frequency of 50 Hz. The speed of recording was conditioned by the rapid reaction of the skin immediately after the source of thermal stimulation was removed.

The research was carried out in accordance with the guidelines for conducting thermovision research in medicine developed by Prof. Ring from the University of Glamorgan [32]. Both the camera, the room, and the patient had to be properly prepared. The tests were performed in an air-conditioned room with a constant temperature of 20 °C. Before starting the measurements, the camera was switched on for about 15 min to achieve thermal stabilization. The tests were performed in the morning, before any hospital procedures. Before the examination, the patient had the examined part of the skin exposed for about 15 min.

To compensate for patient movement, a motion correction technique based on cross-correlation was employed [13]. To enhance the quality of motion compensation, a piece of aluminum foil with a low-emissivity value was attached to the skin as a reference region, which is clearly visible in the thermovision images. A detailed description of the experiment can be found in [13]. Despite the movement correction, the temperature curve is still noisy. Figure 7 displays the visual and thermal images of the patient with psoriasis. The regions of interest for the unhealthy (top) and healthy (bottom) parts of the skin are cooled down and marked on the thermal image. The distance and size of the region of interest (ROI) can influence the temperature value [33]. However, in the presented approach, this factor is not as significant since two ROIs are used for comparison and they are positioned close to each other, ensuring that the camera distance is the same for both measurements. Furthermore, the absolute temperature itself is not crucial; rather, the dynamic reaction of the skin and the difference in obtained time constants for both cases are more important. The primary concern lies in reducing the noise in the obtained signal, which is mainly caused by the movement of the patient, resulting in changes in the ROI’s location. Noise minimization is mainly achieved through movement correction of the marked ROIs. The healthy and unhealthy parts of the patient’s skin for the ROIs were selected to be close to each other in order to cool down and register both regions at the same time. 

**Figure 8:**
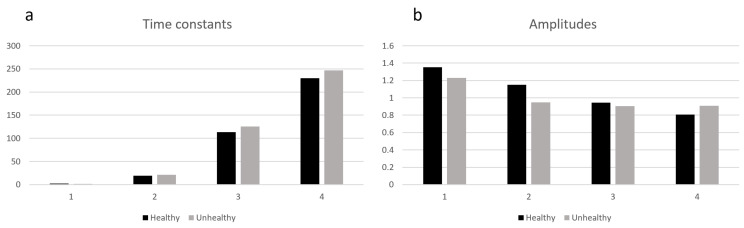
Time constants (**a**) and amplitudes (**b**) for patient’s data.

**Figure 9:**
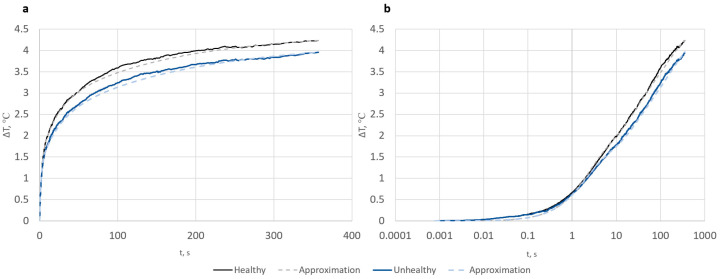
Measurements of temperature in time of healthy and unhealthy skin after thermal excitation and their approximations in linear (**a**) and logarithmic scales (**b**).

The results—the values of predicted time constants and their amplitudes—are presented in Figure 8, while the measurements for healthy and unhealthy (affected by psoriasis) parts of the skin and their approximations are shown in Figure 9.

Finally, the recovered distribution of time constants and their amplitudes are presented in Table 4. As expected, the inflammation area of the skin is responding slowly. The additional thermal inertia is observed there due to the change in vascularization.

The distribution of recovered time constants seems to be accurate. In skin tissue measurements, the range of time constants should differ, as it does in this case. The lowest time constants fall within the range of seconds, while the longest time-constant values reach the level of hundreds of seconds. Such a range distribution is natural and aligns with the fast initial response of the curve and the eventual steady state at the end [30]. 

The correctness of the obtained values was confirmed by the results already published in the literature. In [30], burned skin was modeled as a two-time-constant approximation. The levels of time constants obtained were in the range of tens of seconds for the first and hundreds of seconds for the second.

Time constants are different for healthy and unhealthy parts of the skin. The proposed method could be utilized to differentiate between pathological and physiological tissue states, which would aid in the diagnosis and treatment of skin illnesses like psoriasis.

## 4. Discussion and Conclusions

The article presents a novel CNN-based method for recovering thermal time constants from a temperature–time curve after thermal excitation. The training procedure involved the generation of temperature–time curves and the validation of the results with and without noise. The trained network was then tested using real measurements from a patient suffering from psoriasis.

The presented approach shows a good approximation of the input functions both for the validation data generated artificially with and without noise as well as for data obtained from thermographic temperature measurements over time. The accuracy of the time constants is further supported by the low values of the mean squared error temperature rise in time, which are 0.00316479 for healthy skin and 0.00337056 for unhealthy skin. According to the proposed scaling and normalization of all data to the range [0, 1], as mentioned above, the obtained relative error of the presented method is less than 10%. This indicates that the predicted values are close to the actual measurements, confirming the accuracy of the results.

The CNN training dataset uses scaled inputs, which makes the approximation problem more general. The input data may vary in size and duration, but they are always normalized to the same range. In order to reduce the size of the input data and shorten the learning phase, non-uniform sampling was used. Finally, rescaling the output results with time and temperature allows for the proper distribution of thermal time constants. The proposed CNN uses the weighted loss function for time constants and their amplitudes. Since the influence of the amplitudes of the time constants on the final results is weaker, their contribution to the training was increased.

The results appear to be promising. The regression CNN network shows potential in thermal time-constant spectroscopy, particularly for biomedical thermographic signals that are often noisy and disturbed. The approximation error is sufficiently small to accurately reproduce the original time constants that can be used to distinguish between physiological and pathological tissue states. In addition, compared to other optimization methods, the presented approach consistently gives satisfactory results and avoids the problem of obtaining negative values for *τi*, which can occur when using non-constrained optimization methods such as fminsearch [34]. Despite the ill-conditioned nature of the dynamic thermal system, the CNN works well and provides satisfactory results for input signal parameters varying over wide ranges.

Several improvements could be made, such as testing more complex CNN networks or pre-defined ones such as ResNet, AlexNet, etc. Including noisy data used in the training procedure can also be beneficial. The main challenge lies in determining the number of time constants that appear in the thermal process. The presented approach assumes an approximation of four time constants, but this may not always be sufficient. It is important to collect more measurements to confirm the correctness of the proposed approach and compare it with other methods using the same dataset.

The problem of time-constant spectrometry, especially for thermal processes, is very difficult. This is due to the fact that the exponential functions used to represent the temperature evolution in time are not orthogonal function series. The aim of this paper was to confirm that the CNN approximation can be useful to deal with this problem. The verification of the correctness of using this approach can be performed using other methods that are related to less ill-conditioned optimization problems, such as electromagnetic and electrical network identifications, as in [12]. In addition, novel approaches to CNN network correctness verification can also be applied [35,36,37,38].

## Figures and Tables

**Table 1:** CNN parameters.

Parameter	Values/Method
Exponential parts	4
*τ_i_* range	(0–1) s
*T_i_* range	(0–1) °C
Train dataset/epoch	10,000
Validation data	1000
Optimizer	Stochastic Gradient Decent
Loss Function	0.2·mse(τ) + 0.8·mse(T)
Epoch no.	5000
Activation function	sigmoid

**Table 2:** Mean squared errors estimated by original and predicted data.

Component No.	*τ_i_*	*T_i_*	*T*(*t*)
1	0.00128	0.03642	0.01353
2	0.01022	0.05394	
3	0.01336	0.0555	
4	0.01327	0.05225	
mean	0.00953	0.04952	

**Table 3:** Mean squared errors estimated between original and predicted data for thermal time constants, amplitudes and temperature rise over time at different levels of added noise.

Noise Variance	*i*	*τ_i_*	*T_i_*	*T*(*t*)
0.0001	1	0.00179	0.03289	0.01180
	2	0.01010	0.05359	
	3	0.01468	0.05708	
	4	0.01615	0.04853	
	mean	0.01068	0.04802	
0.0005	1	0.00391	0.04794	0.01261
	2	0.01459	0.06010	
	3	0.02036	0.05894	
	4	0.02189	0.05778	
	mean	0.01518	0.05619	
0.001	1	0.00707	0.06066	0.02051
	2	0.01524	0.06194	
	3	0.02753	0.05982	
	4	0.03403	0.06350	
	mean	0.02096	0.06148	
0.0015	1	0.00930	0.06405	0.01909
	2	0.01818	0.06456	
	3	0.03471	0.06316	
	4	0.03842	0.06611	
	mean	0.02515	0.06447	
0.0025	1	0.00948	0.07460	0.03609
	2	0.02351	0.06238	
	3	0.04262	0.06651	
	4	0.06015	0.07994	
	mean	0.03394	0.07085	
0.005	1	0.01724	0.09785	0.04929
	2	0.02643	0.06824	
	3	0.05885	0.07596	
	4	0.08743	0.07964	
	mean	0.04748	0.08782	

**Table 4:** Recovery time constants and amplitudes for healthy and unhealthy cases.

Case	*τ_i_*	*T_i_*
Healthy	1.9486818	1.354312
	18.936752	1.1490566
	113.21907	0.9453574
	230.25365	0.8074841
Unhealthy	1.7264072	1.231425
	21.461962	0.9482369
	125.83128	0.902929
	246.69447	0.9088539

## Data Availability

The data presented in this study are available on request from the corresponding author.

## References

[1] Marco S., Palacin J., Samitier J. (2001). Improved multiexponential transient spectroscopy by iterative deconvolution. IEEE Trans. Instrum. Meas..

[2] Garnier H., Mensler M., Richard A.A. (2003). Continuous-time Model Identification from Sampled Data: Implementation Issues and Performance Evaluation. Int. J. Control.

[3] Ljung L. Experiments with Identification of Continuous-Time Models. Proceedings of the 15th IFAC Symposium on System Identification.

[4] Yarman B.S., Kilinc A., Aksen A. (2004). Immitance Data Modelling via Linear Interpolation Techniques: A Classical Circuit Theory Approach. Int. J. Circ. Theory Appl..

[5] Jibia A.U., Salami M.J. (2012). An Appraisal of Gardner Transform-Based Method of Transient Multiexponential Signal Analysis. Int. J. Comput. Theory Eng..

[6] De Tommasi L., Magnani A., De Magistris M. (2017). Advancements in the identification of passive RC networks for compact modeling of thermal effects in electronic devices and systems. Int. J. Numer. Model..

[7] Shindo Y., Noro O. (2014). Effective frequency range of ladder network realization for complex permeability of magnetic sheets. IEEJ Trans. Elec. Electron. Eng..

[8] Wang K., Chen M.Z.Q., Chen G. (2017). Realization of a transfer function as a passive two-port RC ladder network with a specified gain. Int. J. Circ. Theory. Appl..

[9] Szekely V. (1991). On the representation of infinite-length distributed RC one-ports. IEEE Trans. Circuits Syst..

[10] Szekely V. (1998). Identification of RC networks by deconvolution: Chances and limits. IEEE Trans. Circuits Syst..

[11] Vermeersch B. (2009). Thermal AC Modelling, Simulation and Experimental Analysis of Microelectronic Structures including Na-Noscale and High-Speed Effects. Ph.D. Thesis.

[12] Gustavsen B. (2006). Improving the pole relocating properties of vector fitting. IEEE Trans. Power Deliv..

[13] Strakowska M., Strąkowski R., Strzelecki M., De Mey G., Wiecek B. (2018). Thermal modelling and screening method for skin pathologies using active thermography. Biocybern. Biomed. Eng..

[14] Strakowska M., Chatzipanagiotou P., De Mey M., Chatziathanasiou V., Wiecek B. Novel software for medical and technical Object Identification (TOI) using dynamic temperature measurements by fast IR cameras. Proceedings of the 14th Quantitative Infra-Red Thermography Conference.

[15] Chatzipanagiotou P., Chatziathanasiou V., Papagiannopoulos I., De Mey G., Wiecek B. (2013). Dynamic thermal analysis of underground medium power cables using thermal impedance, time constant distribution and structure function. Appl. Therm. Eng..

[16] Chatzipanagiotou P., Strąkowska M., De Mey G., Więcek B. (2013). A new software tool for transient thermal analysis based on fast IR camera temperature measurement. Meas. Autom. Monit..

[17] CAPTAIN-Computer-AidedProgramforTime-SeriesAnalysisandIdentificationofNoisySystems. http://www.es.lancs.ac.uk/cres/captain/.

[18] Karimifard P., Gharehpetian G.B., Tenbohlen S. (2013). Localization of winding radial deformation and determination of deformation extent using vector fitting-based estimated transfer function. Euro. Trans. Electr. Power.

[19] Strakowska M., Chatzipanagiotou P., De Mey G., Chatziathanasiou V., Więcek B. (2020). Multilayer thermal object identification in frequency domain using IR thermography and vector fitting. Int. J. Circuit. Theory Appl..

[20] Gupta J., Pathak S., Kumar G. (2022). Deep Learning (CNN) and Transfer Learning: A Review. J. Phys. Conf. Ser..

[21] Kim J.-H., Lee J.-S. Deep Residual Network with Enhanced Upscaling Module for Super-Resolution. Proceedings of the IEEE Conference on Computer Vision and Pattern Recognition (CVPR) Workshops.

[22] Li J., Fang F., Mei K., Zhang G. Multi-scale Residual Network for Image Super-Resolution. Proceedings of the 15th European Conference on Computer Vision.

[23] Bengio Y., Simard P., Frasconi P. (1994). Learning long-term dependencies with gradient descent is difficult. IEEE Trans. Neural Netw..

[24] Glorot X., Bengio Y. Understanding the difficulty of training deep feedforward neural networks. Proceedings of the Thirteenth International Conference on Artificial Intelligence and Statistics.

[25] Ioffe S., Szegedy C. Batch normalization: Accelerating deep network training by reducing internal covariate shift. Proceedings of the 32nd International Conference on International Conference on Machine Learning.

[26] Ornek A.H., Ceylan M. (2023). CodCAM: A new ensemble visual explanation for classification of medical thermal images. Quant. InfraRed Thermogr. J..

[27] Özdil A., Yilmaz B. (2023). Medical infrared thermal image based fatty liver classification using machine and deep learning. Quant. InfraRed Thermogr. J..

[28] Mahoro E., Akhloufi M. (2022). A Breast cancer classification on thermograms using deep CNN and transformers. Quant. InfraRed Thermogr. J..

[29] Bardhan S., Nath S., Debnath T., Bhattacharjee D., Bhowmik M.K. (2022). Designing of an inflammatory knee joint thermogram dataset for arthritis classification using deep convolution neural network. Quant. InfraRed Thermogr. J..

[30] Kaczmarek M., Nowakowski A. (2016). Active IR-Thermal Imaging in Medicine. J. Nondestruct. Eval..

[31] https://www.tensorflow.org/guide/keras?hl=pl.

[32] Ring E.F.J., Ammer K. (2000). The Technique of InfraRed Imaging in Medicine. Thermol. Int..

[33] Machado Á.S., Cañada-Soriano M., Jimenez-Perez I., Gil-Calvo M., Pivetta Carpes F., Perez-Soriano P., Ignacio Priego-Quesada J. (2022). Distance and camera features measurements affect the detection of temperature asymmetries using infrared thermography. Quant. InfraRed Thermogr. J..

[34] Lagarias J.C., Reeds J.A., Wright M.H., Wright P.E. (1998). Convergence Properties of the Nelder-Mead Simplex Method in Low Dimensions. SIAM J. Optim..

[35] Krichen M., Mihoub A., Alzahrani M.Y., Adoni W.Y.H., Nahhal T. Are Formal Methods Applicable to Machine Learning And Artificial Intelligence?. Proceedings of the 2022 2nd International Conference of Smart Systems and Emerging Technologies (SMARTTECH).

[36] Gehr T., Mirman M., Drachsler-Cohen D., Tsankov P., Chaudhuri S., Vechev M. Ai2: Safety and robustness certification of neural networks with abstract interpretation. Proceedings of the 2018 IEEE Symposium on Security and Privacy (SP).

[37] Singh G., Gehr T., Mirman M., Puschel M., Vechev M.T. (2018). Fast and effective robustness certification. NeurIPS.

[38] Singh G., Gehr T., Puschel M., Vechev M. (2019). An abstract domain for certifying neural networks. Proc. ACM Program. Lang..

